# Pore-intrusion of polymeric binder in supercapacitor electrodes decreases capacitance

**DOI:** 10.1039/d5nr05282c

**Published:** 2026-04-30

**Authors:** Julia Im, Xinyu Liu, Christopher A. O'Keefe, Clare P. Grey, Alexander C. Forse

**Affiliations:** a Yusuf Hamied Department of Chemistry, University of Cambridge Cambridge CB2 1EW UK acf50@cam.ac.uk; b Department of Chemical and Biomolecular Engineering, University of California, Berkeley Berkeley California 94720 USA

## Abstract

A combined study of electrochemical characterization, scanning electron microscopy, gas sorption, and solid-state nuclear magnetic resonance spectroscopy was conducted to understand the effect of polymeric binder on the performance of supercapacitor electrodes. We show that increasing the quantity of PTFE binder in the carbon electrode decreases the gravimetric capacitance. The decrease in capacitance is caused by the decrease in porosity of the carbon electrode, as determined by gas sorption and NMR spectroscopy. Importantly, ^19^F NMR reveals the significant intrusion of the PTFE binder into the carbon micropores, evidenced through the observation of a nucleus-independent chemical shift. ^23^Na NMR of aqueous electrolyte adsorption further shows that increasing the quantity of the PTFE binder hinders the amount of Na^+^ ions adsorbed within the pores, affecting the charge storage mechanism. To mitigate this effect, an alternative dry electrode processing method was investigated, which revealed a substantial reduction in PTFE pore intrusion. The reduced pore intrusion leads to excess PTFE accumulating in the intergranular gaps, which diminishes electrochemical performance in aqueous electrolytes. Summarizing, our study reveals the significant intrusion of polymeric binder into the pores of carbon electrodes, which decreases porosity and the corresponding charge storage performance. These findings may guide the design of new electrode formulations, such as a dry process, with improved energy storage capacities.

## Abstract 2

A combined study using electrochemical characterization, SEM, gas sorption, and solid-state NMR spectroscopy reveals significant binder intrusion into the electrode pores, which impacts supercapacitor electrochemical performance.

## Graphical abstract

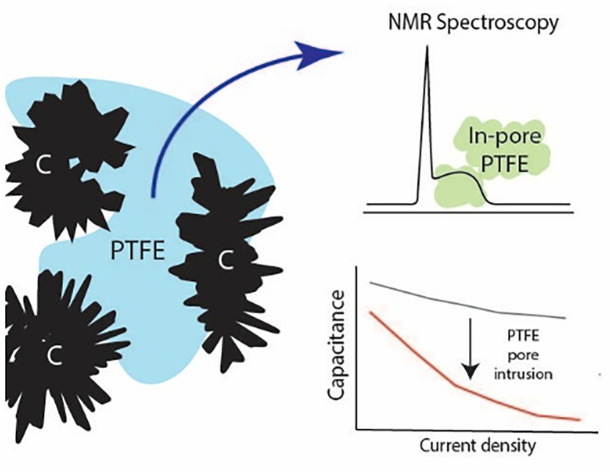

## Introduction

Electrochemical capacitors (ECs) are energy storage systems that can be fully charged or discharged in a very short time, characterized by low energy density and high power density.^1–3^ Electrochemical double layer capacitors (EDLCs) are a class of ECs that use porous carbon electrodes with high surface area, which electrostatically store charge using reversible adsorption of ions from the electrolyte.^1,4,5^ Although EDLCs have attracted attention as devices for use in high-power applications alongside batteries, improvement in the performance of EDLCs is much needed.^1,6–8^ One approach to the improvement of the performance of EDLCs is the choice and design of the electrode materials.^8^ Activated carbons are the most common electrode materials in EDLCs due to their desirable properties, including availability, low cost, non-toxicity, and high chemical stability.^8^ A major focus of the work surrounding the improvement of EDLC electrodes resides in the effort to optimize surface area and porosity.^1,5,8^ A recent study on nanoporous carbon-based electrodes further revealed the importance of structural disorder in improving the capacitance of EDLCs.^9^

An EDLC electrode consists of the active material and a polymeric binder (and in some cases, conductive additives).^4^ The polymeric binder plays an essential role in improving the performance and flexibility of the supercapacitor electrodes and may affect the electrode stability and irreversible capacity losses.^10^ The choice and design of the polymeric binder in the electrode synthesis must meet a few requirements, including mechanical stability, optimized microscopic structure, electrochemical inertness, and low environmental impact.^4^ Studies focusing on the polymeric binders of supercapacitor electrodes typically explore the different potential binder materials.^4,10,11^ In general, two major branches of polymeric binders are suggested: fluorinated and non-fluorinated binders. Carboxymethyl cellulose (CMC) is the state-of-the-art aqueous, non-fluorinated binder, now routinely used in graphitic anodes in lithium-ion batteries, while the more widely used fluorinated, polymeric binders are polyvinylidene fluoride (PVDF), Nafion, and polytetrafluoroethylene (PTFE).^4,10^ In particular, PTFE is the most widely used due to its insulating properties, hydrophobicity, and ability to form fibers, which help hold the electrode structure together.^10^

One way to optimize electrode design is to improve the fabrication process. In particular, previous works have been done on the optimization and implementation of the dry processing method.^12,13^ Differing from traditional methods such as slurry casting, which uses liquid/organic solvents like ethanol, dry processing offers significant advantages such as the elimination of toxic solvent waste and reduced processing cost.^12^ Pameté *et al.* further demonstrated that the dry processing method results in improved electrochemical performance in non-aqueous electrolytes, showing enhanced energy storage capacity, higher energy and power densities, and extended lifespan.^13^

Still, little work has been conducted on the role of the electrochemically inert binders in the supercapacitor field. In particular, there has been a lack of understanding of the effect of polymeric binders in the supercapacitor electrodes, and the dynamics that govern the interaction between the binder and the active materials. In general, the conventional carbon electrode film preparations utilize 5 wt% of binder.^10^ Increasing the binder weight percentage reduces the volumetric capacitance of the electrode since less active material is present in the electrode film. Further, a higher amount of binder incorporation is discouraged because the binder reduces the surface area of the electrode, hindering the ability to store charges, lowering capacitance.^10^ It was further proposed that the binder additives could block the entrances to the pores of the activated carbons, with the different capacitances being observed for electrodes prepared with PTFE and PVDF.^14,15^ Despite this progress, the detailed structure of polymer–carbon composite electrodes and their impact on electrolyte ion adsorption and electrochemical performance remains unclear.

Inspired by recent studies of polymer intrusion into metal–organic frameworks in mixed matrix membranes, we proposed that solid-state nuclear magnetic resonance (ssNMR) spectroscopy could provide new insights into the structures of polymer–carbon composite supercapacitor electrodes.^16–20^ We hypothesized that the use of ssNMR would allow the study of interactions between the polymeric binder, active material, and the electrolyte ions. Specifically, ssNMR can probe the local chemical structure of EDLC electrodes using nucleus-independent chemical shifts (NICS).^9,21^ Molecules or ions close to carbon surfaces give rise to spectral features distinct from more remote species due to the circulation of delocalized π electrons in the sp^2^-hybridized carbon, resulting in ring-current shifts.^21,22^ Here we show that ssNMR spectroscopy can be used to study the structure of polymer-nanoporous carbon composite electrodes, and find evidence for polymer intrusion into carbon pores, which impacts electrolyte adsorption. Combining the results of ssNMR, gas sorption, and scanning electron microscopy (SEM) imaging, we explain the capacitance decreases observed in electrodes at higher binder content. Finally, we show that the dry process method, relative to the slurry-based method, significantly drops the amount of PTFE that intrudes into the carbon micropores. Additionally, while the dry process method effectively prevents PTFE intrusion into the carbon pores, the resulting increase in surface hydrophobicity adversely affects electrolyte wetting and leads to worse electrochemical performance in aqueous electrolytes. These findings highlight that different electrode fabrication methods introduce distinct pore-blocking mechanisms, which affect electrode properties and electrochemical performance. We hope that this work motivates future studies to understand interactions between binder and electrode as a key factor in supercapacitor electrode design.

## Results and discussion

**Fig. 1:**
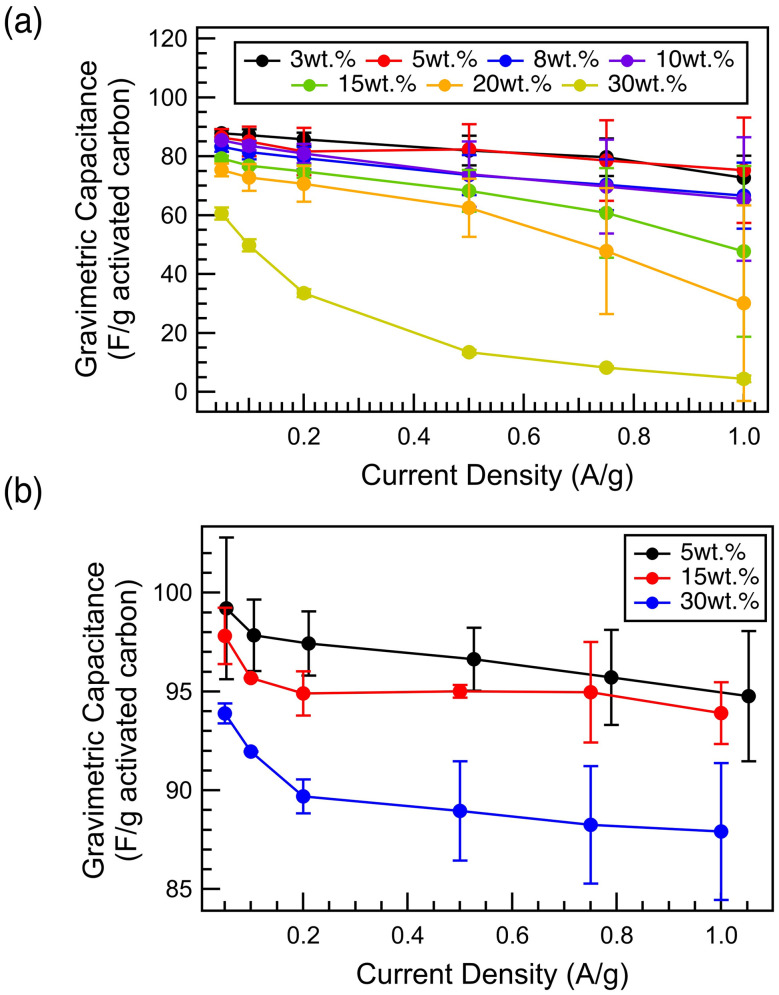
Gravimetric capacitance of the carbon electrode films of varying PTFE binder weight percentages, prepared *via* the wet method. The gravimetric capacitance is calculated based on the capacitance per the mass of activated carbons in each film. Gravimetric capacitance was measured for (a) aqueous cell using 1 M Na_2_SO_4 (aq)_ electrolyte (b) nonaqueous cell using 1 M TEABF_4_ in acetonitrile.

To quantify the impact of the polymeric binder on supercapacitor performance, capacitance measurements were conducted on cells with aqueous electrolytes and YP50F carbon electrodes of varying PTFE content prepared *via* a standard “wet method” (Fig. 1a). Increasing the PTFE binder weight percentage decreases the gravimetric capacitance, agreeing with previous literature.^10^ Our gravimetric capacitance values shown in Fig. 1 are normalized by the mass of activated carbon, rather than the full electrode mass, and so they directly reflect the extent to which the PTFE content impacts the performance of the carbon. Further, the electrode films of higher binder weight percentages experience a steeper downwards slope, *i.e.*, poorer performance at high charging rate. The poorer high-rate performance for films with higher PTFE content indicates limited electrolyte ion diffusion through the films due to PTFE blocking the transport pathways.^23^ In addition to the direct capacitance measurement, we strongly recommend future work to incorporate electrochemical impedance spectroscopy (EIS) and long-term stability study for a full understanding of the electrochemical performance as a function of binder composition.

We test the hypothesis that the decrease in the gravimetric capacitance is not solely due to the hydrophobicity of the PTFE binder by performing identical tests on coin cells with a non-aqueous acetonitrile-based electrolyte (Fig. 1b). Despite the smaller decrease in the observed capacitance with increasing binder content, we still observe a monotonic decrease. This shows that while the hydrophobicity of the PTFE binder does have a significant impact on the electrochemical performance when using the “wet method”, there are further mechanisms that cause the decrease in capacitance as the binder content is increased. Interestingly, the decrease in capacitance in a nonaqueous cell with increasing binder content is more significant in lower current density, where the micropore-driven charge storage (*i.e.*, the number of available sites for binding within the pores) drives the electrochemical performance, rather than the ability to reach the sites (*i.e.*, the tortuosity and pore accessibility).^23^

The interaction between the active materials and the polymeric binder was initially studied using scanning electron microscopy (SEM), a commonly used technique to visualize the microscopic structure and phases of polymers (SI 2).^10,11,24^ Regions of PTFE clustering were observed, showing the affinity of the PTFE binder to adhere to smaller particles and gaps, rather than the smoother surfaces of the bigger particles. These clusters became more concentrated in electrode films with higher PTFE compositions (Fig. S4). This is consistent with a previous study on high-temperature polymer electrolyte fuel cells (HT-PEFCs), where it was found that the increasing PTFE binder weight percentages hinder the homogeneity of the electrode due to the formation of compact PTFE clusters.^25–27^ The lack of ability to homogenize the structure and composition of the electrode is due to the hydrophobicity of PTFE and the inability to create a uniform dispersion.^26^

High magnification SEM images show how the PTFE binder integrates into the gaps of activated carbon particles. With increasing PTFE binder content, the PTFE binder fills up the intergranular gaps and increasingly impacts the film structure and composition (Fig. S4). PTFE binder in the intergranular pores hinders the diffusive transport of ions and charge and is the cause of the steeper slope for the higher binder weight percentage electrode films seen in Fig. 1.^23,27^ The SEM images further show the heterogeneous morphology of PTFE binder within the film, indicating the possibility of diverse pathways of PTFE integration into the activated carbon particles (Fig. S4). Yet, ultimately, SEM gives no direct information on how PTFE might impact the carbon porosity.

**Fig. 2:**
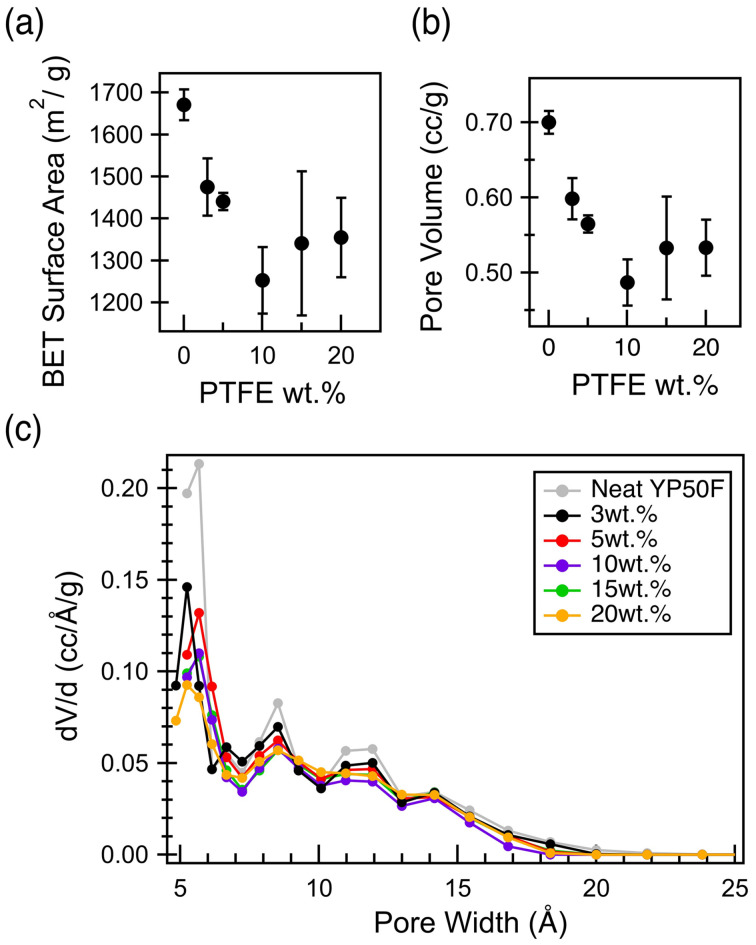
Surface area and pore size distribution for electrode film of varying PTFE weight percentage (prepared *via* the wet method). (a) BET surface area (b) cumulative pore volume calculated from pore size distribution comparing the electrode films to neat, activated carbon powders and (c) pore size distribution calculated based on the basis of quenched solid density functional theory analysis (slit pore model) of N_2_ isotherms at 77 K.

Since the carbon pores are the major contributor to the electrochemical performance of a supercapacitor electrode, it is critical to understand how the incorporation of PTFE binder affects the porosity of the carbon electrodes.^1^ N_2_ physisorption experiments were conducted to quantify both the surface area and porosity of these electrode films in comparison to the neat, activated carbon particles (Fig. S5). With increasing PTFE binder content up to 10 wt%, the BET surface area decreases significantly, along with the cumulative pore volume (Fig. 2a and b), before an apparent plateau is reached. These decreases are explained by the pore size distribution plot, which shows a significant decrease in the microporosity in the electrode films with increasing PTFE content (Fig. 2c). Furthermore, even for the sample with the lowest PTFE wt%, a significant decrease in porosity is observed below 7 Å pore width, compared to the pristine activated carbon particles. Therefore, it is proposed that the decrease in capacitance with increasing PTFE binder weight percentage (Fig. 1) is due to the decrease in porosity of the carbon films (Fig. 2).^10,14,15^

**Fig. 3:**
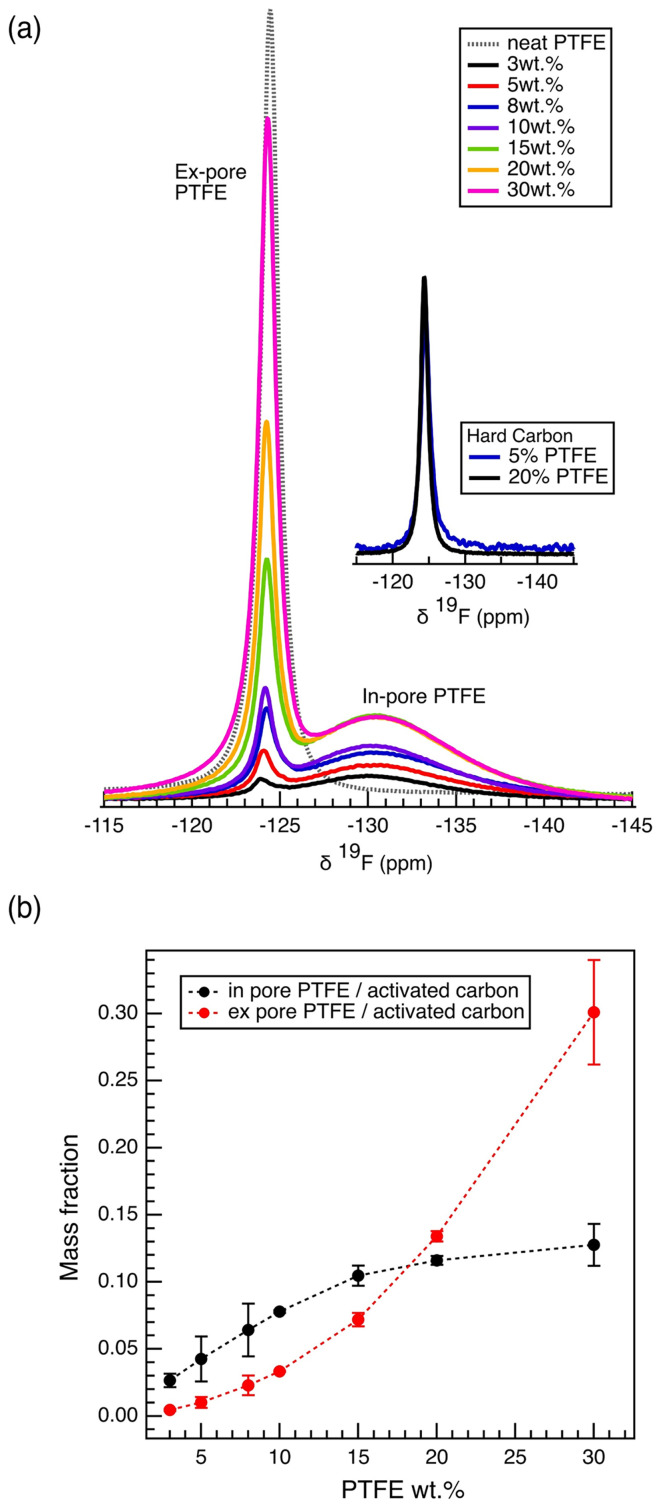
1D ^19^F MAS NMR. (a) ^19^F MAS NMR spectra of the porous electrode films (wet method), normalized with respect to the sample mass. Spectra of the hard carbon, nonporous electrode films (wet method) are also shown in comparison (Fig. S5). (b) calculated in-pore and ex-pore PTFE mass per mass of activated carbon; mass of in-pore and ex-pore PTFE was calculated from the deconvoluted ^19^F spectra and the measured sample mass.

The mechanism by which the binder hinders the porosity of the electrode film was investigated using solid-state nuclear magnetic spectroscopy (ssNMR). The ^19^F magic-angle spinning (MAS) NMR spectrum of the neat carbon electrode films show two distinct spectral features, a broad feature at −130.6 ppm, assigned to PTFE binder located within the carbon micropores (in-pore resonance), and a sharp feature at −124.2 ppm, assigned to PTFE binder located in the intergranular gaps between the carbon particles (ex-pore resonance)^9,21,28–31^ (Fig. 3a). The ex-pore resonance, aligning with the neat PTFE resonance, correlates to the PTFE visible in the SEM images (Fig. S4). The chemical difference between the two resonances, calculated as Δ*δ* = *δ*_in-pore PTFE_ − *δ*_ex-pore PTFE_, is −6.4 ppm. Although no work on polymeric activated carbon complex has shown nucleus-independent chemical shift (NICS), this particular Δ*δ* value has been observed in the past with electrolytes adsorbed in the micropores of the YP50F system.^32^ Further, in computational studies that use Δ*δ* as a tool to probe pore sizes, the chemical shift difference of −6 ppm represents adsorption into micropores of diameter less than 1 nm.^33^

To test the hypothesis that the in-pore resonance originates from the PTFE binder located within the carbon micropores, additional carbon films were produced using the same PTFE binder but with nonporous hard carbon (Fig. 3a). The ^19^F NMR spectra of the films made of nonporous carbon show only one sharp resonance overlapping with the ex-pore peak, while the broad in-pore resonance disappeared, supporting that the in-pore resonance originates from the PTFE located within the carbon micropores, rather than on external surfaces of the primary carbon particles.

The ^19^F MAS NMR spectra allow the quantification of the amount of PTFE located within the carbon micropores (in-pore) and the intergranular spaces (ex-pore). Remarkably, at the lowest PTFE content of 3%, 87.5% of the PTFE is located within the carbon pores, with just 12.5% outside the pores (Fig. S7). With increasing PTFE weight percentage, we observe a decrease in the relative population of the in-pore PTFE (Fig. 3a, Fig. S7). Further, the mass of in-pore PTFE per mass of activated carbon increases logarithmically, reaching a maximum around 0.13 mg in-pore PTFE per mg of activated carbon (Fig. 3b). This plateau reaches the saturation point at which the maximum amount of PTFE has gone into the pores. Thus, it is likely that the significant drop in the capacitance observed in films of high PTFE weight percentages (>15 wt%) is rather affected by the diffusive limit of ions travelling through the PTFE clusters that exist in the intergranular gaps, rather than further pore-blocking effects.

**Fig. 4:**
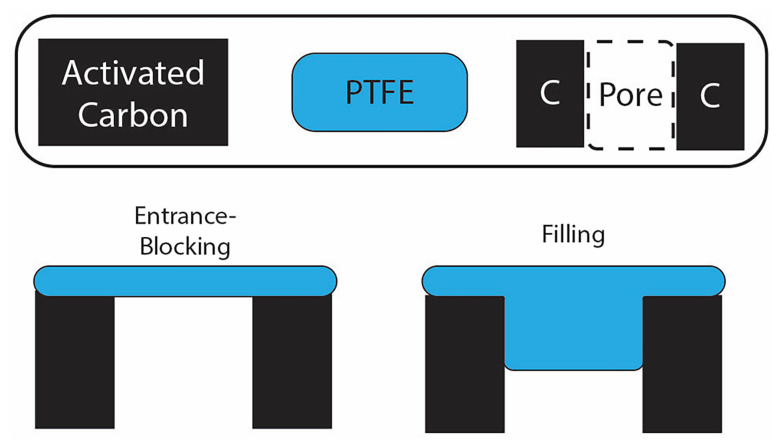
Schematics of mechanisms in which the PTFE binders block the porous network in the activated carbon; the black box represents the activated carbon particle with its carbon layers, the gap between the two blocks being a porous space in between the carbon layers, and the blue box represents the PTFE polymer. The entrance-blocking mechanism is when the PTFE does not actually penetrate the pores, but rather creates an obstruction to entry. The filling mechanism is when the PTFE penetrates into the pores.

Importantly, the largest increase of in-pore PTFE content occurs below 15 wt% (Fig. 3b), mirroring the gas sorption results, which show the largest porosity decreases for samples at low PTFE content (Fig. 2a and b). Further analysis of the results from N_2_ physisorption and ^19^F NMR shows a discrepancy in the quantitative prediction of the amount of PTFE located within the carbon micropores (Fig. S8). Specifically, the decrease in cumulative porous volume as calculated by the pore size distribution from the physisorption data is much greater than the maximum predicted by the ^19^F NMR. This discrepancy brings clarity into the pore-blocking mechanism by the PTFE binder (Fig. 4). The in-pore resonance of the ^19^F NMR spectra proves the filling mechanism in which the PTFE penetrates the porous network of the activated carbon, directly reducing the porosity. The filling mechanism predicts that the PTFE binder physically occupies a sizable portion of the porous volume. However, this mechanism does not fully account for the decrease in porous volume observed by the physisorption data. This discrepancy can be accounted for with the entrance-blocking mechanism, where the PTFE binder sits on top of the pore entrance and transitions the open pore into a closed pore, inaccessible to any fluids. The combination of both mechanisms explains how the PTFE binders decrease the porosity of the activated carbon.

**Fig. 5:**
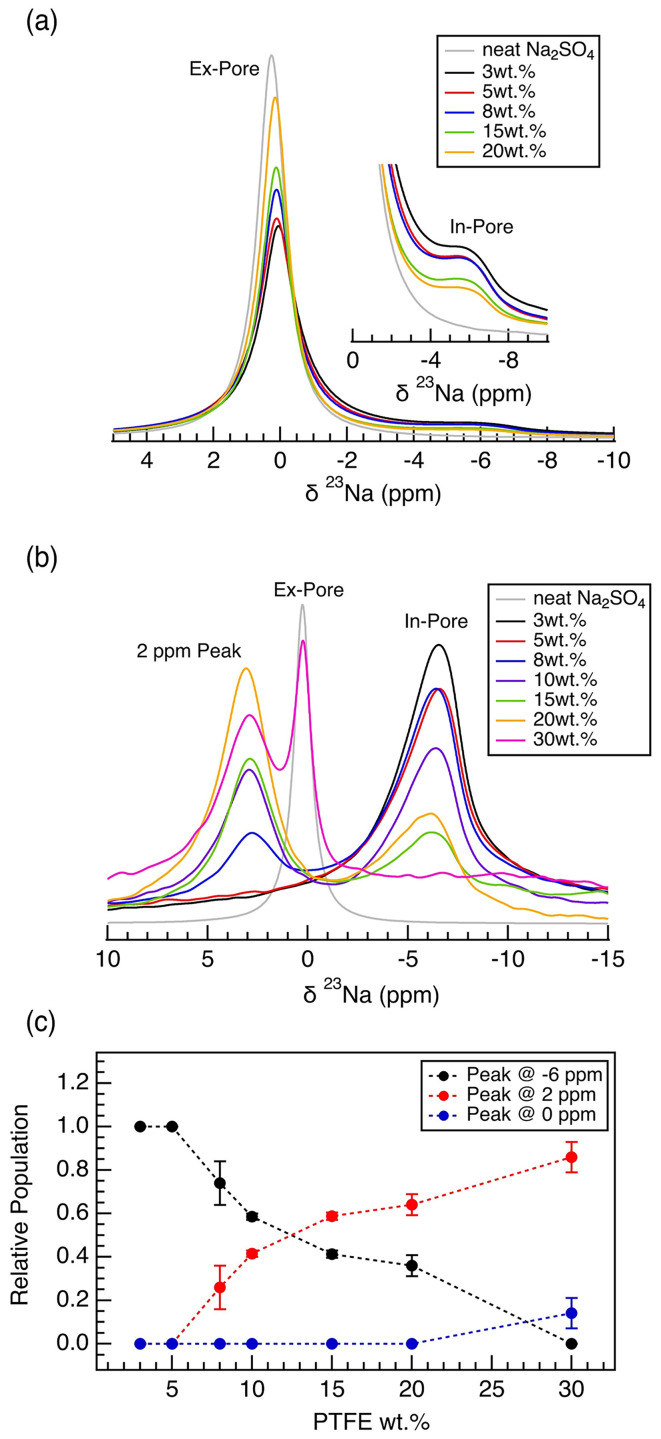
1D ^23^Na MAS NMR on carbon electrode films (wet method) of different PTFE weight percentage soaked in 1 M Na_2_SO_4(aq)_ electrolyte (a) 1.5 : 1 v/w electrolyte to electrode ratio sample prepared in the MAS rotor; magnified view of the −6 ppm region shown in the side (b) 0.5 : 1 v/w ratio sample prepared in the MAS rotor (c) relative population of each peaks (−6, 0, 2 ppm) calculated *via* deconvolution.

The pore-blocking mechanism of the PTFE binder in the carbon electrode films affects the electrochemical performance of the carbon electrode films by hindering the ion adsorption in the carbon electrode. The effect of the binder on the interaction between the electrolyte ions and the carbon was evaluated using an electrolyte ion adsorption study *via*^23^Na MAS NMR spectroscopy, using Na_2_SO_4(aq.)_ as the electrolyte salt. For a 1.5 : 1 v/w ratio of electrolyte to electrode film samples, ^23^Na NMR spectra have two distinct resonances: a broad feature observed near −6 ppm, originating from the Na^+^ ions located within the carbon micropores (in-pore ions), and a sharp feature observed near 0 ppm, originating from the free Na^+^ ions located within the intergranular gaps (Fig. 5a). With increasing PTFE binder content in the carbon electrode films, the intensity of the in-pore resonance decreases due to decreased porosity. Thus, the capacitance of the carbon electrode decreases with fewer ions able to undergo electrosorption within the carbon micropores (Fig. 1).

In the case of a low v/w ratio of 0.5 : 1, the resulting ^23^Na NMR spectra indicate a significant interaction between the PTFE binder and the Na^+^ ions (Fig. 5b). Unlike the spectra with less electrolyte, the NMR spectra are now composed of three distinct resonances, a broad peak around −6 ppm, labelled as in-pore resonance, a sharp peak around 0 ppm aligning with the free electrolyte (ex-pore), and an additional peak located around 2 ppm. A positive shift of the electrolyte resonance (calculated by Δ*δ* = *δ*_in-pore_ − *δ*_neat electrolyte_) has only been reported for MOF-based structure but not in a system composed of activated carbons with dominating ring current effect.^18,34^ Recent work on glass fiber separator reported a downfield shift in 23Na signal in the presence of PTFE nanospheres, consistent with our observation in low v/w electrolyte to electrode samples.^35^ We hypothesize that the 2 ppm resonance arises from the Na^+^ ions that are trapped within the PTFE polymers that exist in the intergranular gaps. The resonance must originate from an environment significantly distant from the bulk electrolyte or near the graphene sheets. The 2 ppm resonance is dominant in the spectra for the electrode films with high PTFE wt%, supporting the hypothesis that the interaction between the binder and the Na^+^ ions is the origin of the 2 ppm resonance (Fig. 5c). Specifically, with increasing binder weight percentage, the polymeric binder located in intergranular gaps is possibly trapping the ions. The reason we do not observe the 2 ppm resonance in high electrolyte loading is due to the abundance of bulk electrolyte. The signal from the bulk electrolyte is extremely strong, possibly overshadowing any remaining intensity from the 2 ppm resonance. Further, with the abundance of aqueous electrolyte, any ions stuck in the intergranular PTFE region are likely mobilized and undergo fast exchange with the bulk electrolyte ions, leading to the loss of the 2 ppm resonance. After all, we emphasize that our explanation of the +2 ppm resonance remains a hypothesis, and future work is needed to validate its origin.

**Fig. 6:**
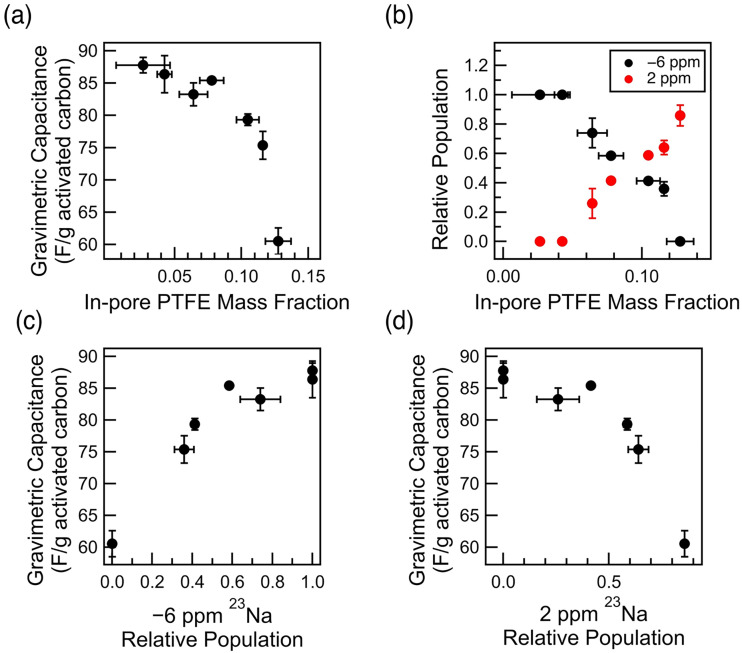
Relationship between the gravimetric capacitance and the quantitative ^19^F and ^23^Na NMR (a) measured gravimetric capacitance for aqueous samples of varying mass fraction of in-pore PTFE per activated carbon (wet method); the gravimetric capacitance is for 0.05 A g^−1^ current density (b) the relationship between the in-pore PTFE mass fraction and the relative Na^+^ ion population from ^23^Na NMR (c) gravimetric capacitance of the aqueous samples compared to the −6 ppm relative Na^+^ ion population (d) gravimetric capacitance of the aqueous samples compared to the 2 ppm relative Na^+^ ion population.

The decrease in capacitance in lower PTFE weight percentage films represents the consequence of the decrease in porosity due to PTFE (Fig. 1). However, the phenomenon observed in the films of higher PTFE weight percentages reflects the consequence of the interaction between the electrolyte and the hydrophobic PTFE binder. This phenomenon explains the more drastic decrease in gravimetric capacitance in aqueous cells, especially with those of higher PTFE wt%. Specifically, the decrease in gravimetric capacitance for aqueous samples with high in-pore PTFE mass fraction experience a much steeper dependence, past the PTFE mass fraction of 0.08, with respect to the mass of the activated carbon (Fig. 6a). The change in in-pore PTFE mass fraction is also associated with the change in the dominating resonance in ^23^Na NMR – a reflection of how the electrolyte ions interact with either the carbon micropores or the PTFE binder. itself.

Correlating the in-pore PTFE mass fraction to the ^23^Na relative population, we observe that mass fraction (mass of PTFE/mass of carbon) of 0.08 marks the point of intersection where the dominating Na^+^ resonance changes from −6 ppm to 2 ppm (Fig. 6b). The steep decrease in gravimetric capacitance in the films of high in-pore PTFE mass fraction (≥0.08) is due to the majority of the Na^+^ ions interacting with the PTFE clusters in the intergranular gaps, rather than locating within the carbon micropores. Particularly, the steep decrease in gravimetric capacitance is observed when −6 ppm ^23^Na relative population is below 0.5, and the 2 ppm ^23^Na relative population is above 0.5 (Fig. 6c and d). Thus, we conclude that the significant exchange between the dominating Na^+^ ions from the carbon micropores to the PTFE cluster in the intergranular gaps is directly impacting the gravimetric capacitance. The decrease in electrochemical performance is a result of decreasing porosity, which results in more Na^+^ ions within the PTFE clusters in the intergranular gaps, rather than in the carbon micropores.

**Fig. 7:**
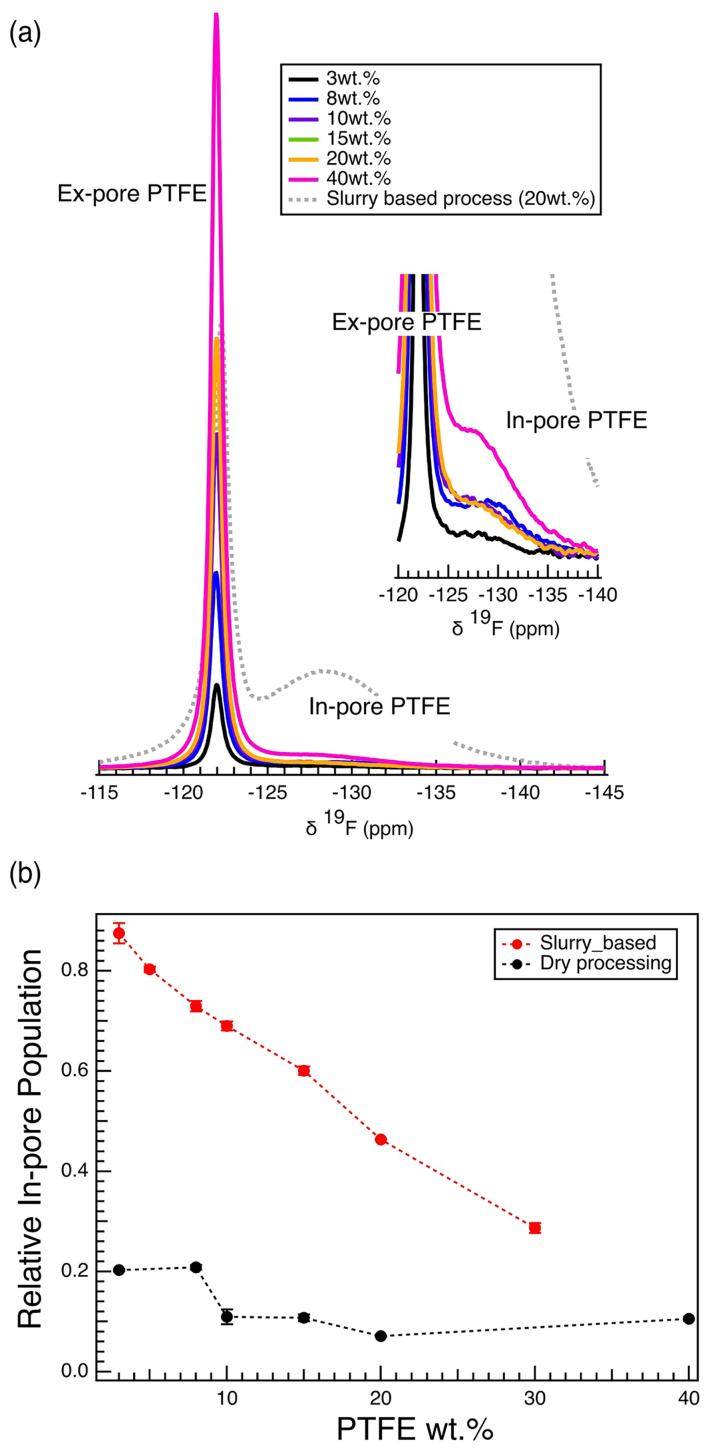
^19^F MAS NMR for dry processed electrode films. (a) ^19^F MAS NMR spectra of the porous electrode films prepared *via* dry processing method, normalized with respect to the sample mass. The in-pore ^19^F region is zoomed in and visualized in the right corner. (b) Calculated in-pore PTFE population relative to the total; the population was calculated from the deconvoluted ^19^F spectra.

The slurry-based wet method, therefore, is subject to high levels of PTFE pore intrusion, which negatively affects the electrochemical performance. We hypothesize that a dry method (SI 1), an energy-efficient process known for yielding long-term cycling performance in lithium-ion battery cathodes,^36–39^ might prevent PTFE pore intrusion. This hypothesis is consistent with recent findings that show improved electrochemical performance in organic electrolytes with the dry-processing electrode fabrication methodology.^13^ Consistent with this, ^19^F NMR results show that the dry method results in significant reduction of the amount of PTFE inside the pores of activated carbon particles (Fig. 7a). For all PTFE wt% samples, the in-pore PTFE resonance (−128.7 ppm) is almost negligible compared to the ex-pore PTFE resonance (−121.9 ppm). In particular, when compared to the 20 wt% electrode fabricated using the wet slurry-based approach, the reduction of in-pore PTFE signal is striking (Fig. 7a). The relative in-pore population, the amount of in-pore PTFE with respect to the total amount of PTFE in the film, shows that only a small fraction (∼15%) of the PTFE goes into the carbon pores (Fig. 7b). This fraction does not change throughout the varying PTFE wt%. This result is different from slurry-based films, in which the relative in-pore population drops with increasing PTFE wt%; this was because the quantity of PTFE pore intrusion is characterized by the mass fraction of PTFE per activated carbon, dictated by the saturation of pores with PTFE (Fig. 3b). This result shows that changing the electrode fabrication method can significantly affect the interaction between the PTFE and the carbon pores. Namely, the dry processing method effectively prevents substantial PTFE pore intrusion by eliminating the liquid carrier (ethanol solvent) that aids the PTFE mobility into these pores. This difference in PTFE pore intrusion between the processing methods is explained by the ease of diffusive transport of PTFE suspension into the pores in the solution state. Due to the linear, unbranched morphology of PTFE, dispersion *via* the slurry method eases the polymer's pore intrusion.^40^

We characterized the dry-processed electrode to evaluate the effect of PTFE pore intrusion on the electrode properties. We report a striking increase in hydrophobicity when using a dry process with PTFE binder (Fig. S9). The change in processing method results in an over 100-degree increase in the contact angle of deionized water. We attribute this effect to residual PTFE binder occupying intergranular gaps, causing the film to be hydrophobic. As a consequence, dry-processed electrodes exhibit poorer electrochemical performance in aqueous electrolytes (Fig. S10). Interestingly, it was previously shown that dry-processed electrodes perform better than slurry-based process when used with non-aqueous electrolytes.^13^ This contrasting behavior across different electrolyte systems highlights the importance of understanding the interaction between the polymeric binder and the active materials in the electrode. Further electrochemical analysis through impedance spectroscopy would help differentiate the slurry-based and dry-processed approach *via* binder-pore interaction. The smoothness of electrodes, as well as varying contributions to resistance, will further clarify how the binder is inhibiting the electrochemical performance.

## Conclusion

In this work, we investigated the effect of polymeric binder on the capacitance of carbon electrodes in supercapacitors. Using electrochemical measurements, we determined that increasing the PTFE weight percentage in the carbon electrode film causes a decrease in gravimetric capacitance. Using a combination of scanning electron microscopy, gas sorption, and NMR, we determined that the decrease in capacitance arises from the decrease in the porosity of the electrode film. This decrease in porosity is, in turn, partially caused by a fraction of the pore space being physically occupied by the PTFE binder, *i.e.*, the PTFE polymer intrudes into the carbon pores. Electrolyte sorption studies using ^23^Na NMR showed how increasing binder incorporation decreases the electrolyte ion adsorption into the carbon pores. Overall, even a minor increase in the amount of PTFE binder in the electrode films significantly hinders the porosity due to significant PTFE intrusion. Alternatively, we show that electrodes fabricated using a dry-processing method prevent PTFE intrusion into micropores. However, the reduced PTFE pore intrusion results in an increase in the film hydrophobicity due to the residual PTFE in the intergranular gaps. This increased hydrophobicity worsens the electrochemical performance of the dry-processed electrodes when used with aqueous electrolytes.

This work offers a comprehensive understanding of the impact of PTFE binder on the capacitance of EDLCs and paves the way for designing electrode formulations with enhanced performance. Our work is the first to show the quantitative and qualitative effects of varying binder composition in the carbon electrode. The use of NMR spectroscopy allows the direct visualization of the chemical environment of the polymeric binder. The direct understanding of the physical mechanism by which the binder blocks the pores of the carbon electrode is important for future design choices. In particular, the increased hydrophobicity of the dry-processed carbon electrode due to the lack of binders in the pores is non-trivial. Further work is needed to address the challenges associated with limiting the intrusion of polymer into the electrode pores as well as its effects on ion transport. In particular, in-depth electrochemical analysis, such as impedance spectroscopy and long-term stability analysis, is needed to characterize the electrochemical performance of the supercapacitor fully.

## Author contributions

J. I.: Data curation, formal analysis, investigation, methodology, visualization, writing – original draft, writing – review & editing. X. L.: Data curation, formal analysis, investigation, methodology, resources, writing – review & editing. C. O.: Investigation, methodology, resources, supervision, writing – review & editing. C. G.: Investigation, resources, supervision, writing – review & editing. A. F.: Conceptualization, funding acquisition, investigation, resources, supervision, writing – review & editing.

## Conflicts of interest

There are no conflicts to declare.

## Supplementary Material

NR-018-D5NR05282C-s001

## Data Availability

The data supporting the findings of this study are available within the article and supplementary information (SI). Supplementary information: methodology, S/TEM images, nitrogen isotherm data, NMR data. See DOI: https://doi.org/10.1039/d5nr05282c. All raw experimental data files are available in the Cambridge Research Repository, Apollo. https://doi.org/10.17863/CAM.117803.
