# Supplementary material for: Pore-intrusion of polymeric binder in supercapacitor electrodes decreases capacitance
Source: Nanoscale. 2026 Apr 30;18(22):11880–9. doi: 10.1039/d5nr05282c (PMC13150818; doi:10.1039/d5nr05282c)
Supplement: NR-018-D5NR05282C-s001 [file NR-018-D5NR05282C-s001.pdf]

Supplementary Information (SI) for Nanoscale.
This journal is © The Royal Society of Chemistry 2026

Supplementary Information:

Pore-intrusion of Polymeric Binder in Supercapacitor Electrodes Decreases Capacitance

Julia Im[1,2], Xinyu Liu[1], Christopher A. O'Keefe[1], Clare P. Grey[1], Alexander C. Forse[1]

[1]Yusuf Hamied Department of Chemistry, University of Cambridge, Cambridge CB2 1EW, UK

[2]Department of Chemical and Biomolecular Engineering, University of California, Berkeley, Berkeley, California 94720, United States

Supplementary Information 1. Methodology....2
Supplementary Information 2. S/TEM Images....5
Supplementary Information 3. Nitrogen Isotherm Data....7
Supplementary Information 4. NMR Results....8
Supplementary Information 5. Contact Angle Measurement....10
Supplementary Information 6. Gravimetric capacitance measurement of the dry-processed electrode....11
Supplementary Information 7. Electrochemical data: Cyclic Voltammetry (CV) and Galvanostatic Charge-Discharge Curve (GCD)....12

Supplementary Information 1. Methodology

Electrode Preparation

Wet method: YP50F activated carbon powders (Kuraray Chemical, Japan) and polytetrafluoroethylene binder (Sigma-Aldrich, 60 wt.% dispersion in water) of varying ratios were mixed into a slurry using ethanol. The particle size of the YP50F activated carbon powders is expressed via 1.9 µm D_{10}, 5.6 µm D_{50}, and 9.7 µm D_{90}. The slurry was then kneaded and rolled to give a free-standing carbon film of approximately 0.25 mm thickness. The films were then stored in a vacuum oven at 90 ºC for a minimum of 48 hours before any measurements were performed. The resulting mass of each carbon film was 200 mg, with the PTFE wt.% ranging from 3 wt.% to 30 wt.%.

Carbotron hard carbon (ANR Technologies) and polytetrafluoroethylene binder (Sigma-Aldrich, 60 wt.% dispersion in water) were used for electrode films of nonporous hard carbons. The D_{50} particle size of the Carbotron hard carbon, as provided by the manufacturer, is 9 µm.[21]

Dry method: YP50F activated carbon powder and PTFE powder (Sigma-Aldrich) of varying ratios were combined on a heated mortar and pestle at 100 °C. For a minimum of 30 minutes, the powders were then ground by hand into a free-standing carbon film; they were then kneaded multiple times to achieve a homogenous distribution of PTFE. The resulting mass of each carbon film was 100 mg, with the PTFE wt.% ranging from 3wt.% to 40wt.%.

Electrochemical Capacitance Measurement

Disc-shaped electrodes were cut using a ¼ inch diameter stainless-steel manual punching cutter, with the two electrodes in each cell having identical mass (masses were within 0.2 mg of each other). The mass range of the carbon film electrodes was 4.7 mg to 8.8 mg. Two-electrode, symmetric 2032-coin cells were fabricated using a Whatman glass fiber separator and 200 µl of electrolyte. The aqueous cells were constructed using 1 M Na_2SO_4 $_{(aq)}$, and the nonaqueous cells were constructed using 1 M $TEABF_4$ in acetonitrile. The nonaqueous cells were constructed in a nitrogen glovebox, with the oxygen and water levels kept below 0.1 ppm. All coin cells were supplied by Cambridge Energy Solutions.

All electrochemical measurements were conducted in a two-electrode configuration with a Biologic BCS-805 potentiostat. Cyclic voltammograms of the cells were obtained at a scan rate of 10 mV/s with a potential window of 0-0.8 V for at least 30 cycles. For constant current charge-discharge measurements, each cell was measured under different current densities at 0.05, 0.1, 0.2, 0.5, 0.75, and 1 A/g with a voltage window of 0-0.8 V for at least 3 cycles under each current density. The current used for the measurement was calculated using the average mass of the active material in both electrodes in the cell. For each PTFE weight percentage, a minimum of three coin cells were constructed. The total capacitance of each cell was calculated using the average galvanostatic charge-discharge measurements from the slope of the discharge curve.[22] The specific

capacitance was calculated by dividing the total capacitance by the mass of the active material. The error bars correspond to the standard deviation between the three cells.

NMR Experiments

The sample preparation for ^{19}F ssNMR experiments was done with an electrode film dried in a vacuum oven for a minimum of 48 hours before the measurement. The films were cut and packed into 4.0 mm zirconia rotors, with a film sample weighing around 40 mg. The mass of the film inside the rotor was determined during packing and unpacking of the rotor and was recorded to yield an average. The experiments were carried out on a Bruker Avance IIIHD spectrometer equipped with an 11.7 T magnet ($\nu_0(^1H)$ = 500.13 MHz, $\nu_0(^{19}F)$ = 470.59 MHz, $\nu_0(^{23}Na)$ = 132.29 MHz). A Bruker triple channel 4 mm MAS probe was used, and experiments were conducted using a sample spinning speed of 14 kHz. Recycle delays were set to allow quantification (i.e., at least 5 times the longest spin-lattice relaxation time (T_1)), around 12 seconds for all samples. ^{19}F NMR samples were referenced relative to neat PTFE at –124.2 ppm. The baseline for the ^{19}F spectra was corrected using an iterative baseline correction algorithm[23], and the deconvolution was performed using the TopSpin SOLA line shape analysis module. The error bars were determined via repeating the line shape analysis three times and taking the standard deviation of iterations.

The ^{19}F ssNMR experiments on dry-processed films were performed using a 9.4 T Bruker BioSpin spectrometer equipped with an Avance IV Neo console with a 1.3 mm double resonance HX magic angle spinning (MAS) probe. The samples were spun at 50 kHz. Recycle delays were set to allow quantification, around 30 seconds for all samples. ^{19}F experiments were acquired using a rotor-synchronized Hahn echo sequence. Spin-spin relaxation (T_2) was measured and used to account for T_2 losses before quantification. DMFit software was used to deconvolute the spectra and process the quantitative information.[24] ^{19}F chemical shifts were referenced with respect to LiF (^{19}F = 204 ppm).

To perform an ion-adsorption study using ^{23}Na ssNMR, a measured amount of film electrode was packed into the rotor so that each rotor contains 20 mg of activated carbon. 10 µl of $Na_2SO_{4\ (aq)}$ electrolyte was added into the rotor for a 0.5:1 electrolyte volume to carbon mass (v/w) loading ratio. Before the ^{23}Na ssNMR experiments, the rotor was sealed with the cap and was left for 24 hours to equilibrate. Following, an additional 20 µl was added to the first set of samples, and the same procedure was repeated for a 1.5:1 v/w ratio. The ^{23}Na ssNMR was conducted using the same spectrometer used for ^{19}F ssNMR. Recycle delays were set to 1 second, and the sample spinning speed was 5 kHz to minimize the disturbance of the adsorbed electrolytes.

Gas Physisorption Experiments

A high vacuum physisorption/chemisorption analyzer (Autosorb iQ from Anton Paar) at 77 K was used for N_2 physisorption measurements. Each carbon film was placed in a glass cell and degassed at 120 ºC for 16 hours before the measurement. Brunauer-Emmett-Teller (BET) surface area was

calculated from isotherms using the BET equation and Rouquerol's consistency criteria implemented in AsiQwin.[25] Pore size distributions were calculated by quenched solid-density function theory (QSDFT) based on a slit pore model.[26]

Electron Microscopy

Scanning electron microscopy (SEM) images were obtained using a TESCAN MIRA3 FEG-SEM equipped with an Oxford Instruments Aztec Energy X-maxN 80 EDS detector (Oxford Instruments, Abingdon, UK). The sample was placed on an adhesive carbon mounted on an Al pin stub.

Transmission electron microscopy (TEM) was obtained using a JEOL JEM-3010 microscope (JEOL, Akishima, Japan). TEM images were recorded using a Gatan 794 CCD camera and analyzed with ImageJ software.

Contact Angle Measurement

A contact angle goniometer (DSA25S, Kruss) was used to analyze the surface wettability of carbon electrodes with DI water.

Supplementary Information 2. S/TEM Images

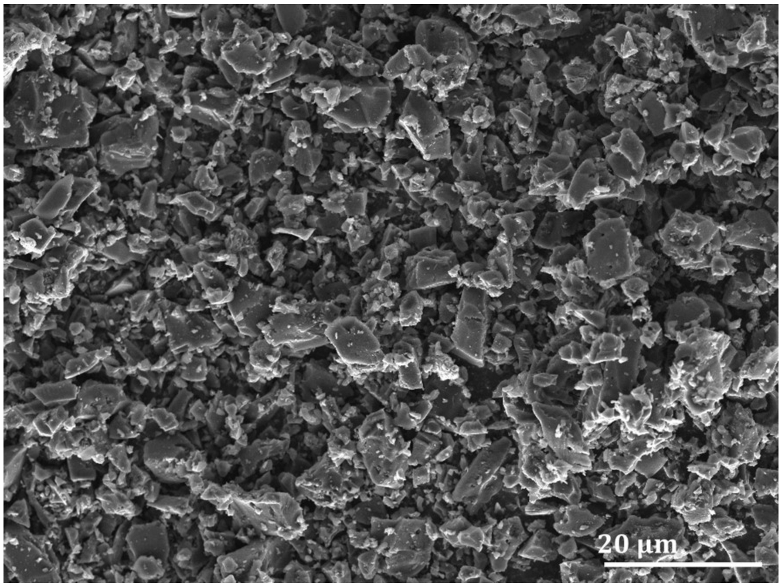

Figure S1. SEM images of the neat YP-50F Activated Carbon powders. Heterogeneity of the activated carbon powders is observed. The activated carbon powders are formed of smaller particles of surface diameter of ~ 1µm and bigger particles of surface diameter of ~ 10 µm with smoother surfaces. The smaller particles tend to conglomerate around the bigger, smoother particles.

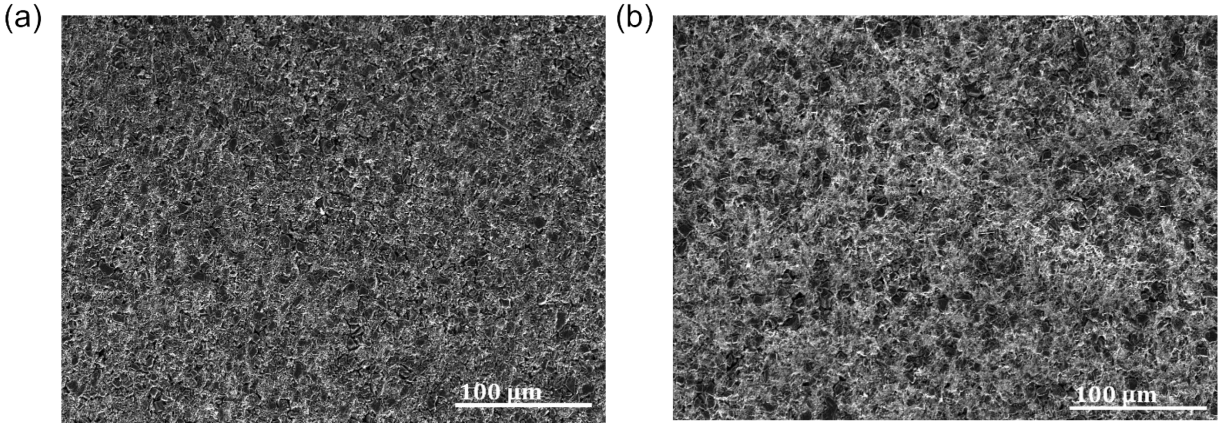

Figure S2. Low magnification SEM images of (a) 10wt.% PTFE electrode film (b) 20wt.% PTFE electrode film; Area of lighter shade in (b) represents regions of cluster, showing the affinity of the PTFE binder to surround the smaller particles and gaps, rather than adhering to the smooth surface of bigger carbon particles.

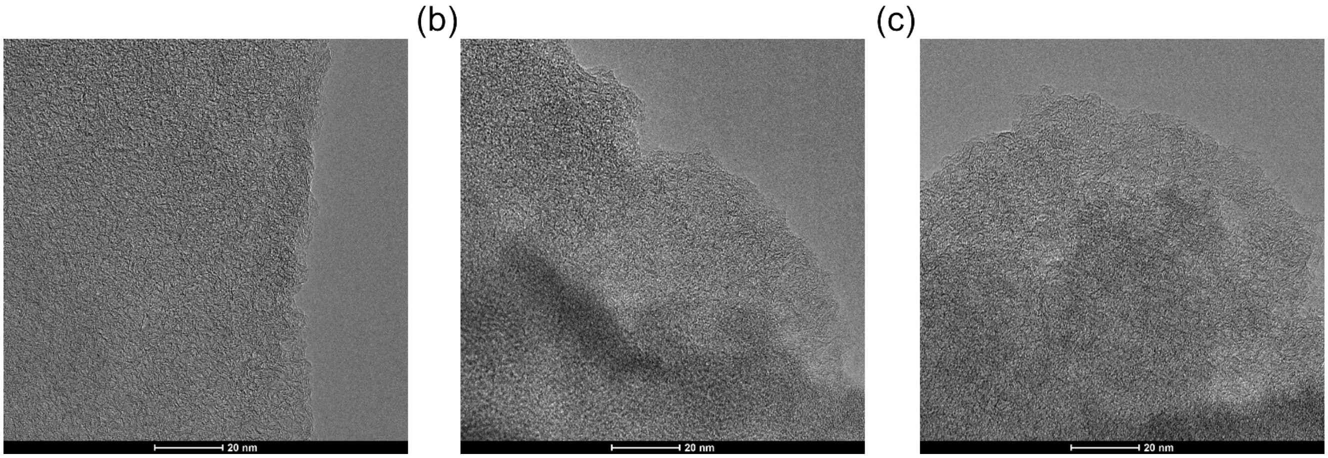

Figure S3. TEM images of (a) neat YP-50F activated carbon (b) 3wt.% PTFE electrode film (c) 20wt.% PTFE electrode film; white regions in the TEM images represent the porous area of the material, which is present for all neat activated carbon particles, low binder weight percentage electrode film, and high binder weight percentage electrode film.

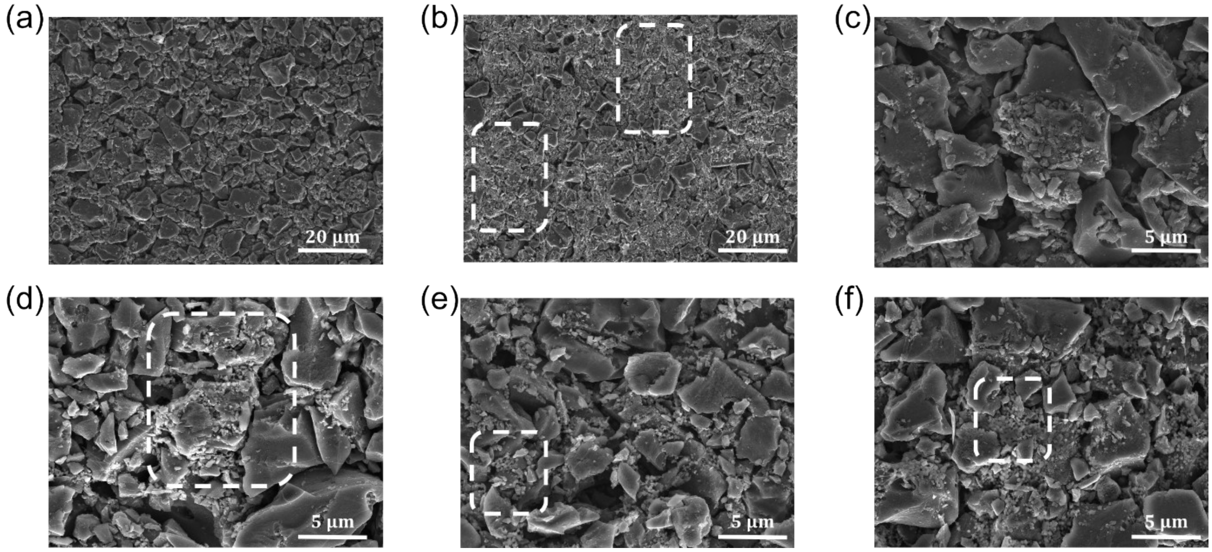

Figure S4. SEM images for the carbon-PTFE electrode with differing binder weight percentages (prepared via the wet method). Low magnification images of the electrode with weight percentages (a) 5 wt.% (b) 20 wt.%; the white box highlights the region of high-density region affected by the PTFE binder; High magnification images of electrodes with weight percentages (c) 3 wt.% where no PTFE is visible (d) 5 wt.%, in which the PTFE binders are integrated in big cracks of the carbon powders (white box) (e) 15 wt.% with the white box highlighting PTFE binder connecting smaller powder to the bigger, smoother particles (f) 20 wt.%, the white box highlighting big gaps filled with PTFE binder

Supplementary Information 3. Nitrogen Isotherm Data

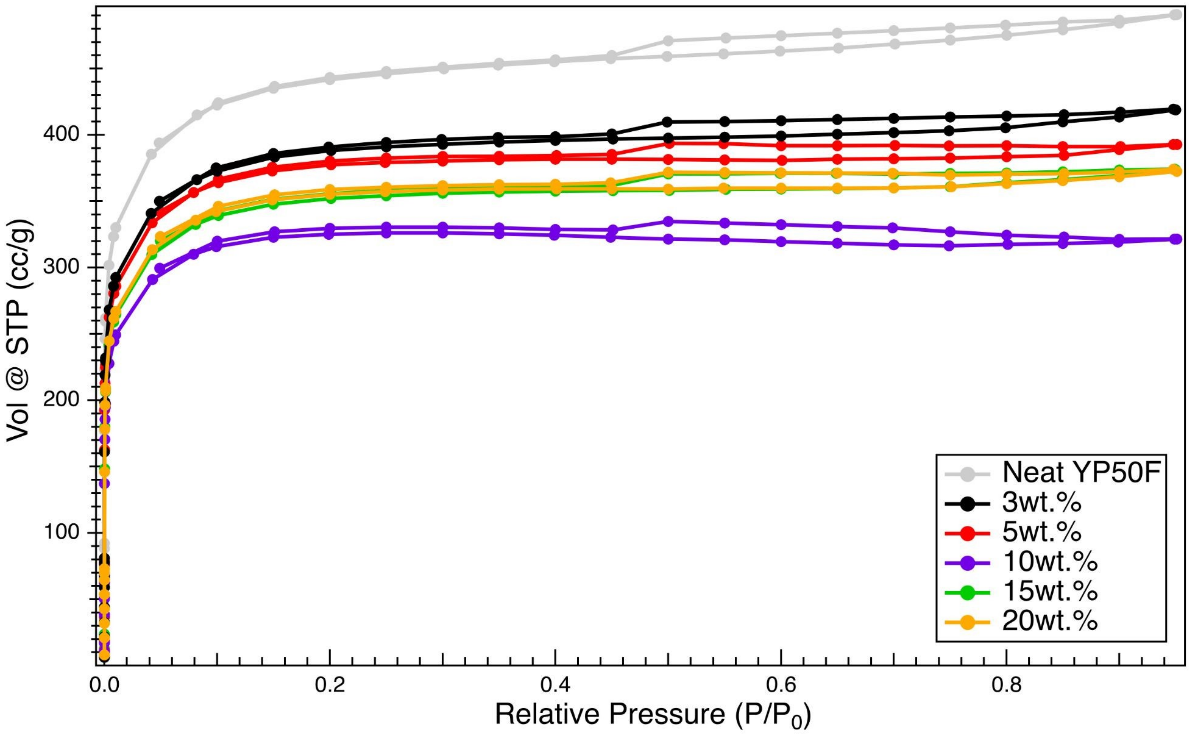

Figure S5. N_2 isotherms measured at 77K for neat YP-50F activated carbon and the electrode films of different PTFE binder weight percentages.

Supplementary Information 4. NMR Results

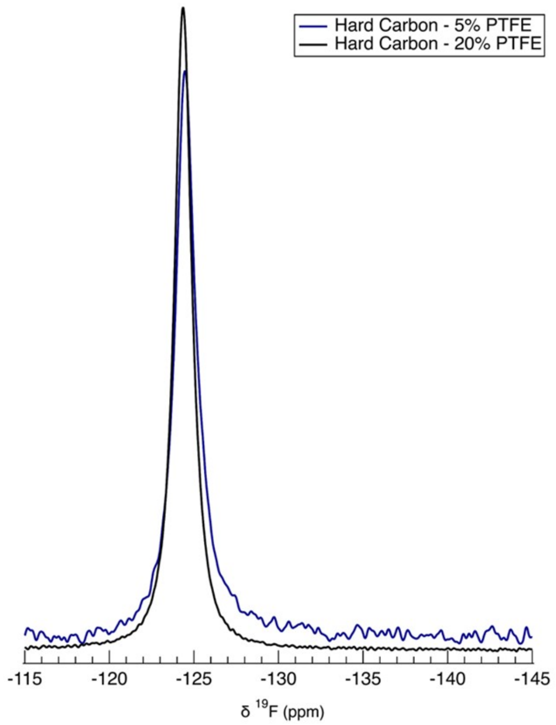

Figure S6. 1D ^{19}F MAS NMR on carbon electrode films made with PTFE aqueous dispersion mixed with non-porous hard carbon with two different binder weight percentages. The spectra show only one resonance that aligns with the free PTFE dispersion and is missing the broad resonance that is classified as in-pore resonance.

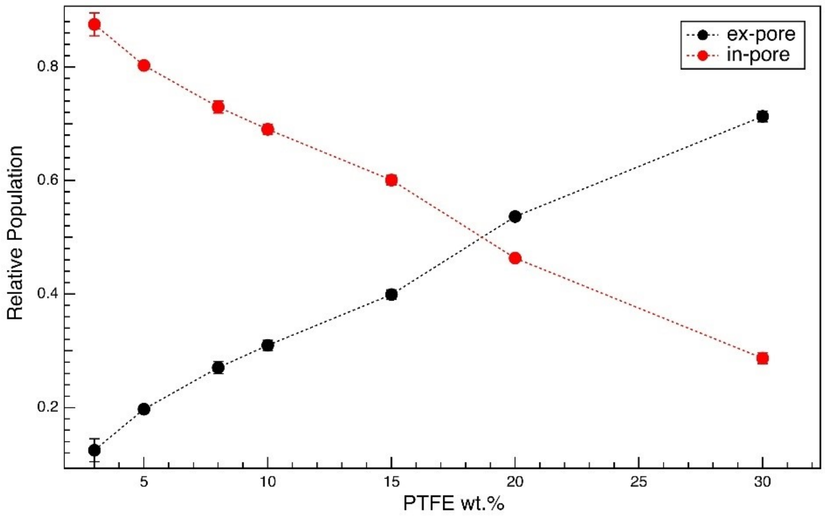

Figure S7. Analysis of 1D ^{19}F spectra of electrode film samples. Calculated relative population from the deconvolution of ^{19}F spectra in Figure 4a. The relative population of in-pore PTFE decreases linearly with increasing PTFE wt.%.

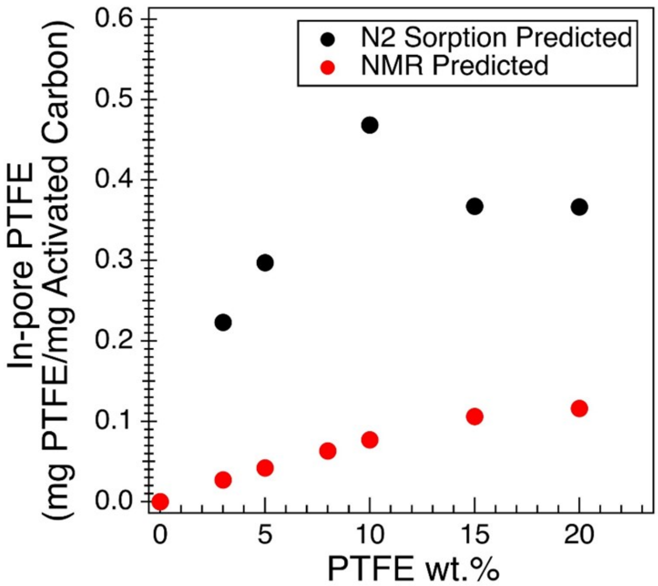

Figure S8. Quantitative prediction of the amount of PTFE that is located within the carbon micropores compared to the amount predicted by NMR in Figure 4b. The N_2 sorption predicted value is calculated by multiplying the decrease in porous volume from Figure 3b, assuming that PTFE retains its bulk density of 2.2g/cc when in nanoconfinement. The NMR predicted value is calculated via quantification of the relative in-pore population via integral of the deconvoluted ^{19}F spectra, followed by multiplying the relative population by the known amount of PTFE in the samples.

Supplementary Information 5. Contact Angle Measurement

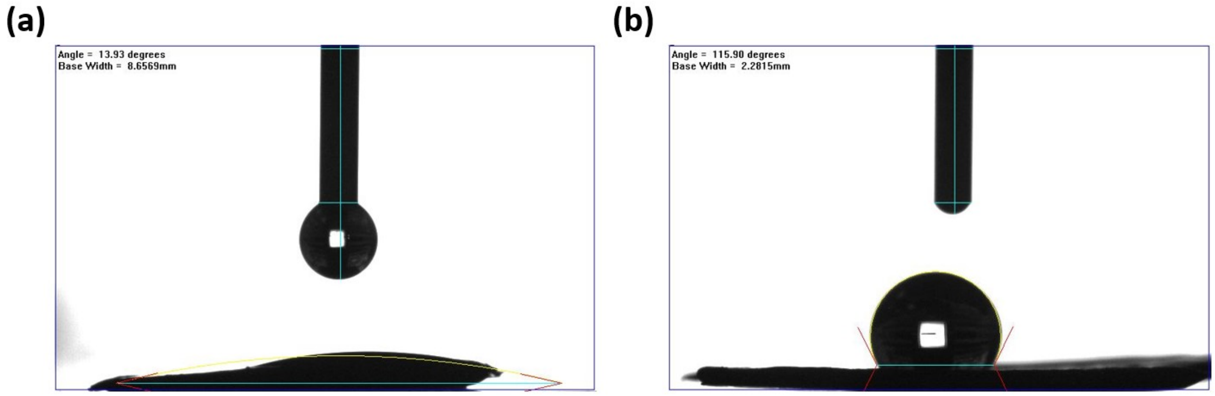

Figure S9. Contact angle measurement data for wet and dry processed electrode, measured with DI water. (a) contact angle measurement of slurry-based electrode with the contact angle of 13.93 degrees (b) contact angle measurement of dry-processed electrode with the contact angle of 115.90 degrees.

Supplementary Information 6. Gravimetric capacitance measurement of the dry-processed electrode

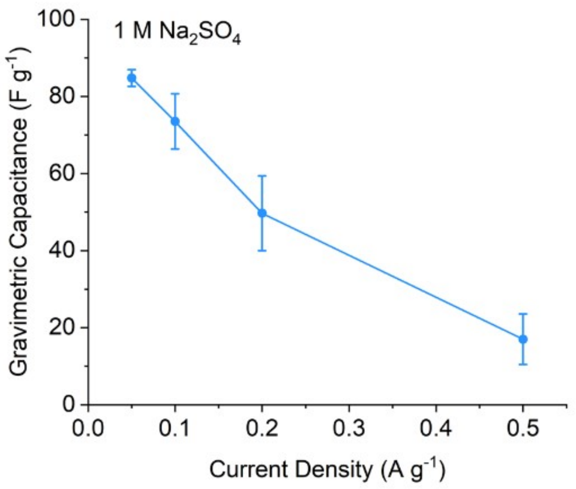

Figure S10. Gravimetric capacitance measurement of the dry-processed electrode with 5 wt.% PTFE binder using 1M Na_2SO_4 (aq) electrolyte. The error bars represent the standard deviations of three repeat cells.

Supplementary Information 7. Electrochemical data: Cyclic Voltammetry (CV) and Galvanostatic Charge-Discharge Curve (GCD)

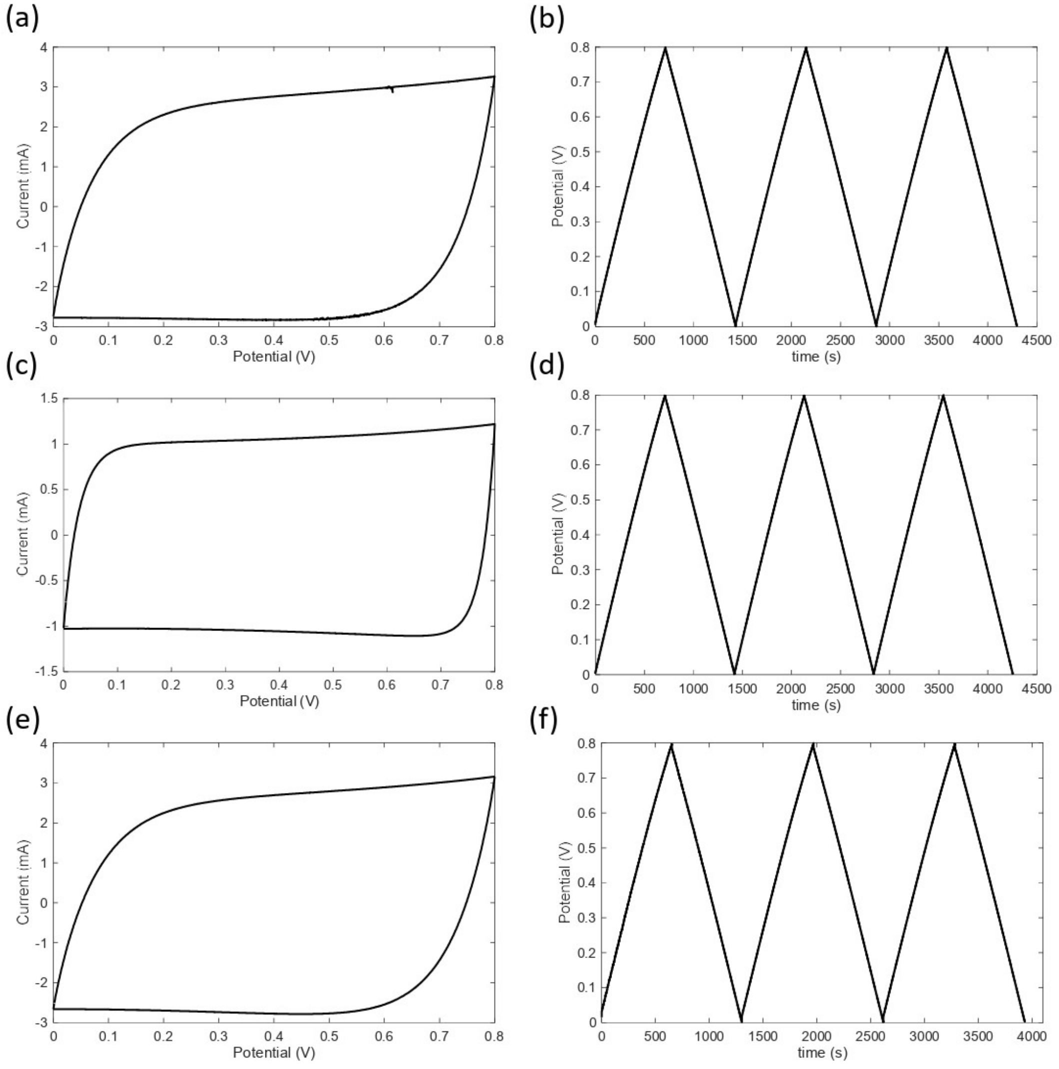

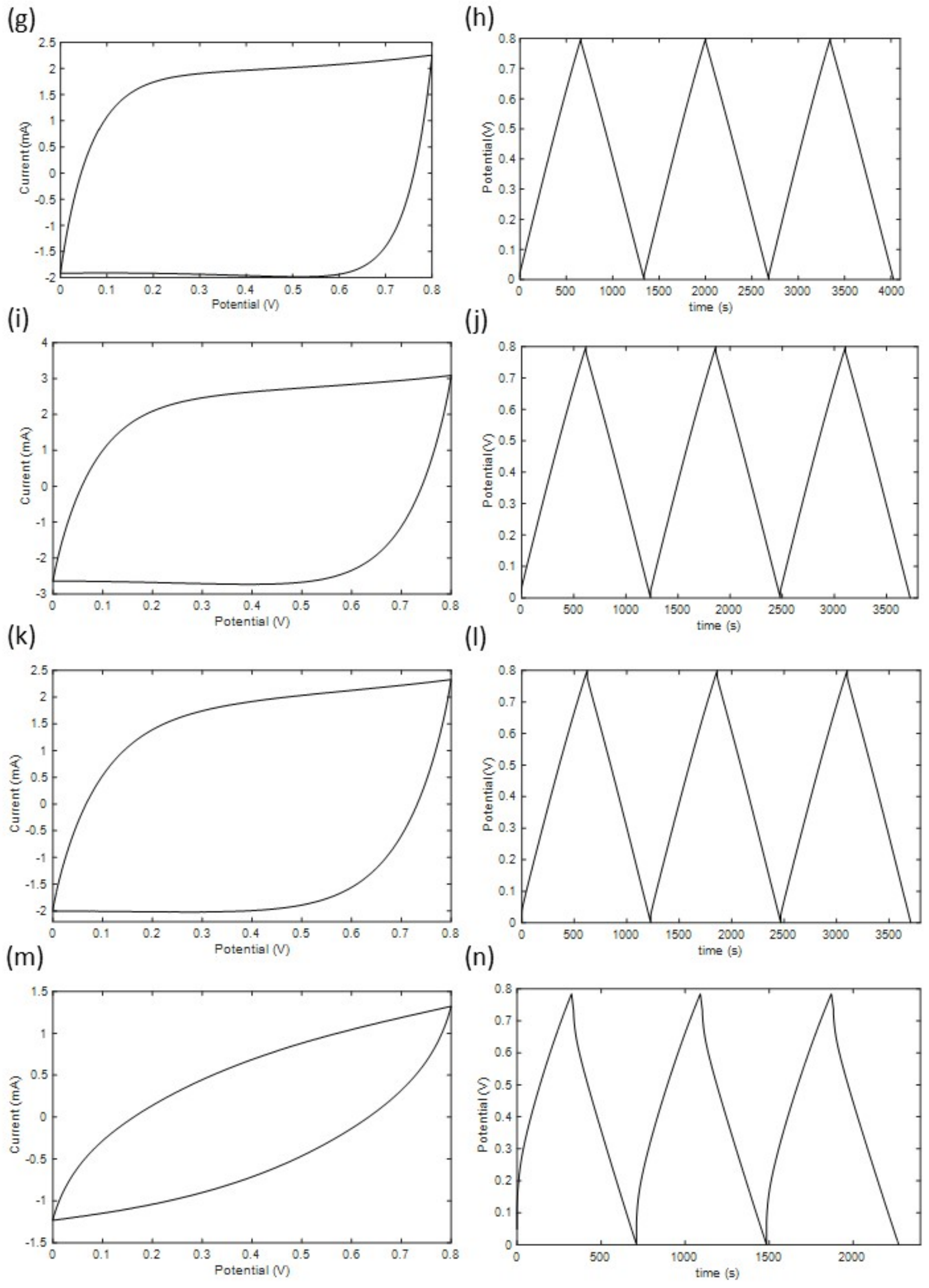

Figure S11. Cyclic voltammetry (CV) and galvanostatic charge-discharge (GCD) curves for samples with binder compositions of 3wt.% ((a) CV, (b) GCD), 5wt.% (c, d), 8wt.% (e, f), 10wt.% (g, h), 15wt.% (i, j), 20wt.% (k, l), and 30wt.% (m, n) binder composition. For each PTFE content, a representative cell from the corresponding batch was selected.
